# Effects of Conventional and Ultrasound-Assisted Washing on Pesticide Residue Reduction and Quality Characteristics of Purslane (*Portulaca oleracea* L.): An Artificial Surface Contamination Study

**DOI:** 10.3390/foods15183317

**Published:** 2026-09-19

**Authors:** Yağmur Küçükduman, Özge Taştan Ülkü, Buket Şahyar

**Affiliations:** 1 Department of Food Engineering, Faculty of Engineering and Natural Sciences, Yeditepe University, Ataşehir, 34755 İstanbul, Turkey; yagmur.kucukduman@yeditepe.edu.tr; 2 Department of Biotechnology, Graduate School, Yeditepe University, Ataşehir, 34755 İstanbul, Turkey; 3 R&D Center, Işık Tarım Ürünleri San.ve Tic. A.S., Kemalpaşa, 35733 İzmir, Turkey; buket.sahyar@isiktarim.com

**Keywords:** pesticide residue reduction, purslane, ultrasound-assisted washing, postharvest decontamination, QuEChERS, LC–MS/MS, GC–MS/MS, quality

## Abstract

Purslane (*Portulaca oleracea* L.) is a nutritious leafy vegetable rich in phenolic compounds, vitamins, minerals, and dietary fiber. However, pesticide residues remaining after cultivation may pose risks to human health, making effective post-harvest decontamination treatments necessary. This study investigated the effects of different washing treatments 5% sodium bicarbonate (SB), 5% acetic acid (AA), 5% citric acid (CA), a mixture containing 2.5% acetic acid and 2.5% citric acid (AA-CA), tap water washing (TW), and ultrasound-assisted washing for 5, 10, and 15 min (US-5′, US-10′, and US-15′, respectively) on pesticide residue reduction and quality characteristics of purslane. This study used an artificial surface contamination model with a 4 h holding period before washing. Residue analyses of acetamiprid, cypermethrin, imidacloprid, and propargite were performed by LC-MS/MS and GC-MS/MS using the QuEChERS extraction method. The antioxidant capacity and total phenolic content of the SB sample were significantly lower than those of the other treatments. Color analysis showed significantly higher ΔE* values in the SB sample. Acidic treatments generally resulted in higher TA% and lower pH and chlorophyll contents than the other treatments. Depending on the pesticide and washing treatment, residue reduction efficiencies ranged from 6.33% to 72.22%. Ultrasound-assisted washing (UAW) provided a favorable balance between residue reduction and preservation of several quality attributes, including total phenolic content (TPC), antioxidant capacity, total flavonoid content (TFC), color, and chlorophyll contents in purslane. These results indicate that ultrasound-assisted washing, particularly the 5 min treatment, provided a favorable balance between pesticide residue reduction and preservation of several quality attributes; however, longer ultrasound exposure was associated with deterioration of some quality characteristics, particularly firmness and weight loss.

## 1. Introduction

Purslane (*Portulaca oleracea* L.), which has been consumed as a traditional medicine and food since antiquity, ranks 8th among the most prevalent plants worldwide [1,2]. In addition to being nutritionally rich in vitamin C, minerals, vitamin E, and ω-3 fatty acids, especially α-linolenic acid, purslane also includes bioactive phytochemicals (phenolic antioxidants and carotenoids, etc.) that are thought to have health benefits [3]. Several biological activities such as antidiabetic, anticancer, bactericidal, diuretic, antiseptic, anti-inflammatory, neuroprotective, antihyperlipidemic, hepatoprotective and anti-arthritic have been attributed to purslane consumption [4]. Purslane is valued for its aromatic taste, similar to spinach, and is a staple of the Mediterranean diet, particularly in Türkiye and Greece [5].

Pesticides are a variety of chemical compounds used to protect the yield and quality of crops and food, to aid in reducing several human diseases caused by insect or rodent vectors, and to destroy pests in agriculture and homes [6]. To meet the food demands of a growing global population and minimize postharvest losses, it is predicted that pesticide use will increase from year to year [7]. However, usage of pesticides has several negative effects on humans and the environment. Excessive pesticide use may contribute to soil degradation, agroecosystem pollution, groundwater contamination, risks to human and animal health, pesticide-residue problems, and the development of resistance in target organisms [8]. Acute illnesses such as eye irritation, headache, convulsions, nausea, diarrhea, respiratory distress and death have also been observed in individuals exposed to high pesticide levels [9]. According to previous research, an average of 30% of fruits and vegetables contain pesticides [10]. The permitted amount of pesticide residues in food is legally controlled by determination of maximum residue levels (MRLs). To minimize consumers’ exposure to harmful or unnecessary pesticide residues, various regulatory agencies restrict the amounts and types of pesticides permitted in food, including through maximum residue limits (MRLs) [11]. Pesticides exhibit a range of physicochemical characteristics and distinct mechanisms of action [12].

The efficacy of washing depends on several factors, including water solubility, hydrophobicity, formulation, penetration into the cuticle, systemic behaviour, time after application, and the characteristics of the plant surface. Dissociation constant (p*K*_a_) and the logarithm of octanol-water partition coefficient (log *P*) of systemic pesticides, like acetamiprid, boscalid, or difenoconazole, are used to assess their physical and chemical features. Some systemic pesticides, including acetamiprid and imidacloprid, are relatively hydrophilic. Systemic pesticides can move through the xylem or phloem transport tissues in vascular plants [13]. Many non-systemic pesticides are relatively lipophilic and may remain associated with the plant surface or cuticular wax layer [14]. Non-systemic pesticides tend to persist on the fruit’s surface; they may be more readily removed by surface-washing treatments [12]. Penetration of most fungicides/insecticides into the cuticle layer of plants is significantly constrained. Therefore, residues of these substances are separated from the fruit after some process such as peeling. For example, peeling fruits like avocados and bananas removes almost all residues from the fruit [15]. However, leafy vegetables such as purslane, lettuce, spinach, and parsley lack a removable peel and are commonly consumed whole. Therefore, pesticide residue poses a significant risk in these types of products.

Processing operations such as cleaning, washing, blanching, peeling, juicing, boiling, grinding, pasteurization, canning, and baking can reduce pesticide residue levels [16]. The washing is a common household practice and can be performed using tap water [17]. The type of produce, pesticide physicochemical properties, contact time, water solubility, and washing treatment can influence washing effectiveness [18,19]. A further factor influencing the elimination of pesticide residue is the concentration of non-toxic chemical solutions [19]. Washing sanitizers are regulated by the US Food and Drug Administration (FDA) as food additives; they are generally recognized as safe (GRAS) in certain situations, such as those involving organic acids and sodium carbonate [18]. In a study conducted on four different pesticides, acidic washing treatments (acetic and citric acid) were found to be more influential than alkaline ones (sodium chloride and sodium carbonate) during pesticide reduction and pesticide reduction was linear with increasing washing concentration. However, the quality characteristics of the product were not examined [20].

Ultrasound-assisted washing (UAW) has been investigated as a potentially time- and energy-efficient approach for pesticide residue reduction [9,21,22]. UAW may reduce pesticide residues without requiring the addition of chemical washing agents. Commercially available versions of this technology have been developed for household use [23]. In a study where different pesticide reduction methods were applied to strawberry samples, it was found that the UAW reduced residues for all pesticides examined with reductions of up to 91.2% [24]. After applying ultrasonic treatment for 20 min, it was discovered that the residual levels of organophosphorus pesticides on raw cucumbers were significantly decreased, reaching as high as 85% [25]. In addition, ultrasonic treatment combined with immersion solutions effectively removes pesticides from lettuce, achieving maximum reduction levels above 80% [26].

Although numerous studies have investigated pesticide reduction in different fruits and vegetables [27,28,29], to the best of our knowledge, the combined effects of conventional and ultrasound-assisted washing on pesticide residues and multiple quality attributes of purslane have not been systematically evaluated. Therefore, the novelty of the present study lies in the comparative assessment of several commonly applicable washing treatments (SB, AA, CA, AA-CA, and TW) alongside ultrasound-assisted washing at different durations (US-5′, US-10′, and US-15′) in purslane, considering both pesticide residue reduction efficiency and changes in physicochemical quality characteristics. It was hypothesized that the extent of pesticide residue reduction would depend on both pesticide physicochemical properties and washing conditions, and that short-duration ultrasound-assisted washing could provide a favorable balance between residue reduction and quality preservation compared with concentrated acidic or alkaline solutions.

## 2. Materials and Methods

### 2.1. Sample Collection and Preparation

Fresh purslane (Portulaca oleracea) was purchased from a local grocery store in Ataşehir, İstanbul, Türkiye. Yellow, visibly damaged, or decayed leaves were discarded. The samples were rinsed with tap water to remove adhering soil and foreign matter, drained for 15 min, and gently blotted with paper towels. The edible leaves and stems were divided into 100 ± 1 g portions. Within each independent batch, the portions were randomly assigned to the contamination and washing treatments. Before artificial pesticide contamination, an untreated subsample from each group was analyzed to confirm the absence or initial concentration of acetamiprid, cypermethrin, imidacloprid, and propargite. The remaining samples were assigned randomly to the contamination and washing treatments. All groups were stored in low-density polyethylene bags in the refrigerator at 4 °C and 95% relative humidity (RH) for no longer than 6 h.

### 2.2. Chemicals and Reagents

QuEChERS kits were obtained from Chromascience (Istanbul, Türkiye). Formic acid (1%), ammonium formate (5 mM), and acetonitrile (100%) were purchased from Merck (Darmstadt, Germany). Certified pesticide standards [Acetamiprid (HPC Standards, 99.90%), cypermethrin (HPC Standards, 98.10%), imidacloprid (HPC Standards, 99.70%), propargite (HPC Standards, 99.10%)] were obtained from Skygen (Moscow, Russia). Phenolphthalein, sodium nitrite and sodium hydroxide were purchased from Riedel-De Haen (Seelze, Germany), Supelco (Bellefonte, PA, USA) and Prolab (Laval, QC, Canada), respectively. Other chemicals were obtained from Sigma Aldrich (St. Louis, MO, USA). All chemicals used without further purification and they were analytical grade.

### 2.3. Pesticides Selection

Pesticides vary widely in their physical and chemical properties, which is an important consideration for regulatory purposes. Acetamiprid, cypermethrin, imidacloprid, and propargite were selected to represent pesticides with markedly different physicochemical characteristics, chemical classes, and systemic behaviors. Acetamiprid and imidacloprid are systemic neonicotinoid insecticides characterized by relatively low log *P* values, whereas cypermethrin and propargite are non-systemic and substantially more hydrophobic (Table A1).

Acetamiprid and imidacloprid are relatively water-soluble neonicotinoid insecticides, whereas cypermethrin and propargite are more hydrophobic compounds with relatively low water solubility. These differences were expected to influence their retention on purslane surfaces and their responses to washing. In addition, acetamiprid, cypermethrin, imidacloprid, and propargite, which were selected as target pesticides, are often found in leafy vegetables. In addition, regulatory relevance was also considered, and the applicable maximum residue levels (MRLs) are presented in Table A1. Pesticide-monitoring studies in Türkiye have additionally demonstrated the occurrence of pesticide residues in vegetables, with acetamiprid reported among the frequently detected compounds [11]. The relevant physicochemical properties, systemic behavior, analytical platforms, and MRLs of the selected pesticides are summarized in Table A1.

### 2.4. Preparation of Standard Solutions

The pesticide working solution was prepared from individual stock solutions (2000 mg/L) of four pesticides. An aliquot of 100 µL from each individual stock solution was combined to obtain an intermediate mixed standard solution containing 200 mg/L of each pesticide. This mixture was subsequently diluted with acetonitrile containing 1% (*v*/*v*) acetic acid to prepare a 20 mg/L mixed solution and finally a 5 mg/L mixed working solution, corresponding to 5 mg/L of each individual pesticide in the mixture. The working standard solution was stored at −20 °C. For the spiking experiments, 2.0 mL of the 5 mg/L mixed working solution was applied to 100 g of purslane samples. Therefore, each sample received 10 µg of each pesticide (40 µg total pesticides), corresponding to a fortification level of 0.10 mg/kg for each analyte. All dilutions and spiking solutions were prepared using acetonitrile containing 1% (*v*/*v*) acetic acid. Using the working standard solutions, matrix-matched calibration standards were prepared by serial dilution with acetonitrile containing 1% (*v*/*v*) acetic acid. For each pesticide, four concentration levels were selected within their respective linear working ranges to construct the calibration curves [30].

### 2.5. Preparation of Contaminated Purslane Samples

After cleaning the purslane samples, a volume of 2.0 mL of the pesticide solution (5 mg/L) was applied uniformly to each 100 g purslane portion using a calibrated micropipette. During application, the leaves were gently mixed to improve the uniformity of pesticide deposition. Then, samples were kept for 4 h at room temperature to allow the deposited solution to dry and interact with the leaf surface. Solvent-control samples were treated with 2.0 mL of pesticide-free application solvent under identical conditions. The initial pesticide concentration (C_0_) was determined separately for each independent experimental batch immediately after the 4 h holding period.

### 2.6. Washing Treatments of Contaminated Purslane Samples

Each 100 g purslane portion was immersed in 400 mL of washing solution, corresponding to a sample-to-solution ratio of 1:4 *w*/*v*. The conventional treatments consisted of tap water, 5% *w*/*v* sodium bicarbonate, 5% *v*/*v* acetic acid, 5% *w*/*v* citric acid, and a mixture containing 2.5% *v*/*v* acetic acid and 2.5% *w*/*v* citric acid (Table 1).

Ultrasound-assisted washing was performed in an ultrasonic bath (DT 510 H, Bandelin electronic GmbH & Co. KG, Berlin, Germany) operating at 35 kHz in continuous mode. The nominal ultrasound power information was 160 W and bath-volume-based power density of 32 W/L. The internal bath dimensions were [30 cm× 24 cm × 15 cm], and the bath was filled with 5 L of water to a depth of 8 cm. Each 100 g purslane portion was immersed in 400 mL of tap water in a glass beaker positioned above the transducers. The bath was degassed for 5 min before treatment. Samples were sonicated in continuous mode for 5, 10, or 15 min. The temperature kept constant between 20 and 25 °C ± 2 °C using a digital thermometer (TFA Dostmann, TA288, Wertheim-Reicholzheim, Germany) during the 5, 10 and 15 min treatment. The temperature was controlled indirectly by adding ice to the bath water.

Untreated samples and only pesticide inoculated samples were used as control. After washing treatments, samples were removed from solutions, and the excess surface liquid was gently removed using clean paper towels. All groups were stored in low-density polyethylene bags in the refrigerator (4 °C, 95% RH) until processing and analysis.

### 2.7. The Pesticide Extraction Process for LC-MS/MS and GC-MS/MS

Briefly, 15 g of homogenized purslane was weighed into a 50 mL polypropylene centrifuge tube. Then, 15 mL of acetonitrile containing 1% acetic acid and QuEChERS extraction salts (1.5 g sodium acetate and 6 g magnesium sulphate) were added into the tube. The mixture was shaken for 1 min using a vortex mixer (Dragon Lab, MX-S, Beijing, China). The mixture was then centrifuged (Sigma, 3–30 K, Osterode am Harz, Germany) at 5000 rpm for 4 min at 4 °C. Approximately 8 mL of the supernatant was transferred and subjected to the QUECHERS clean-up kit (1200 mg magnesium sulphate + 400 mg PSA) (Agilent Technologies, Santa Clara, CA, USA). The mixture was shaken for 1 min by using a vortex. The mixture was subsequently centrifuged at 5000 rpm for 4 min at 4 °C. The supernatant was filtered through a 0.20 µm membrane filter and transferred to a vial. After these procedures, the resulting extracts were analyzed by LC-MS/MS and GC-MS/MS routine pesticide analysis [30].

### 2.8. LC-MS/MS and GC-MS/MS Analysis

LC-MS/MS and GC-MS/MS analyses were performed according to the method described by Taştan et al. [30], with slight modifications.

Acetamiprid and imidacloprid were analyzed using a Shimadzu LCMS-8060 triple-quadrupole mass spectrometer coupled to an HPLC system (Shimadzu Corporation, Kyoto, Japan). Chromatographic separation was achieved on a GL Sciences analytical column (100 mm × 4.6 mm, 3 µm particle size; GL Sciences, Tokyo, Japan) maintained at 40 °C. The mobile phase consisted of 1 mM ammonium formate and 0.1% (*v*/*v*) formic acid in water (Phase A) and 0.1% (*v*/*v*) formic acid in acetonitrile (Phase B). The gradient elution was operated at a constant flow rate of 0.70 mL/min with an injection volume of 5 µL and a total run time of 20 min. The gradient program was applied as follows: 20% B (held for 0.20 min), linearly increased to 95% B at 13.00 min, ramped to 99% B between 13.20 and 17.00 min, and returned to the initial condition of 20% B at 17.01 min for column re-equilibration. Mass spectrometric detection was performed using an electrospray ionization source operating in both positive and negative modes (ESI+/ESI−). The source-dependent parameters were set as follows: ion-source temperature, 300 °C; desolvation temperature, 526 °C; desolvation gas flow rate, 10 L/min; and collision gas pressure, 270 kPa.

Cypermethrin and propargite were analyzed using a Shimadzu GCMS-TQ8040 NX triple-quadrupole gas chromatograph–mass spectrometer (Shimadzu Corporation, Kyoto, Japan). Chromatographic separation was performed on an InertCap 5MS/Sil capillary column (30 m × 0.25 mm I.D., 0.25 µm film thickness; GL Sciences, Tokyo, Japan) using helium as the carrier gas at a constant flow rate of 1.50 mL/min. Samples were introduced in splitless mode with an injection volume of 2 µL at an injector temperature of 250 °C. The GC oven temperature was initially set at 120 °C (held for 2 min), ramped at 20 °C/min to 300 °C, and held at 300 °C for 4 min. The transfer-line and ion-source temperatures were maintained at 250 °C and 230 °C, respectively. The ionization modes, MRM transitions, products, precursors and collision energy (CE) for LC-MS/MS and GC-MS/MS were given in Table A2.

### 2.9. Method Validation

The QuEChERS method was validated in-house according to the SANTE/11312/2021 guidelines [31]. Matrix-matched calibration curves were prepared at four concentration levels by plotting the relative instrument response against the corresponding pesticide concentrations.

Method recovery and precision were evaluated by spiking blank purslane samples with the target pesticides at a fortification level of 0.10 mg/kg in four replicates. The recoveries (%) and repeatability (RSD, %) values were calculated to assess the trueness and repeatability of the method. The obtained recovery and RSD values complied with the acceptance criteria specified in the SANTE guideline [31].

Method sensitivity was evaluated by determining the limits of detection (LOD) and quantification (LOQ) for each target pesticide. Calibration solutions covering the linear working range were analyzed using LC-MS/MS and GC-MS/MS, and the resulting calibration curves exhibited good linearity, with correlation coefficients (R^2^) greater than 0.99. The LOD and LOQ values were calculated based on the linear regression approach using the equations LOD = 3.3 Sa/b and LOQ = 10 Sa/b, where Sa represents the standard deviation of the intercept and b corresponds to the slope of the calibration curve [30,32]. The calculated LOD and LOQ values for all pesticides are presented in Table 2.

Analytical quality control and method validation procedures for pesticide residues analysis applied according to SANTE guideline [31].

The analytical performance of the method was further evaluated in terms of linearity, accuracy, precision, recovery, LOD, and LOQ values as shown in Table 2. Recovery and accuracy values demonstrated the suitability of the method for the determination of acetamiprid, cypermethrin, imidacloprid, and propargite residues in purslane samples.

### 2.10. Calculation of Pesticide Residue Reduction

Formula (1) was used for the calculation of pesticide residue reduction:

(1) $$Pesticide\;residue\;reduction\;(\%)\;=\;[(C_{0}-C_{t})\;/\;C_{0}]\;\times \;100$$

where C_0_ and C_t_ are pesticide residues for initial and after washing treatment, respectively.

### 2.11. Evaluation of Quality Characteristics of Fresh Purslane After Washing Treatments

Cleaned purslane samples were immersed in 400 mL of the washing solutions listed in Table 1. The sample washed only with tap water for 5 min was used as common conventional washing control/reference treatment for all groups. After washing treatments, samples were removed from solutions, and the excess surface liquid was gently removed with clean paper towels. All groups were stored in low-density polyethylene bags in the refrigerator (4 °C, 95% RH) until processing and analysis.

#### 2.11.1. Color and Appearance

After washing, surface color was measured using a colorimeter (Konica Minolta, CM-5, Sakai, Japan). Color parameters (L*, a*, b*) were measured and recorded for all groups. L*, a*, and b* represent lightness, redness, and yellowness, respectively. All tests were performed in duplicate [33]. ΔE* is the index of the total color change in the samples compared with the control sample. It was calculated using Equation (2) [34].

(2) $${\Delta E}^{*}=\sqrt{({{\Delta L}^{*})}^{2}+({{\Delta a}^{*})}^{2}+({{\Delta b}^{*})}^{2}}$$

#### 2.11.2. Titratable Acidity (TA), pH and Soluble Solid Content

Following the washing treatments, an Ultra-Turrax homogenizer (Daihan Scientific Co., HG-15 A, Wonju, Republic of Korea) was used to homogenize 10 g of treated purslane samples with 40 mL of distilled water at 6000 rpm for two minutes. A pH meter (MeterLab, PHM210, Villeurbanne, France) was used to measure the pH of the samples, and the results were recorded. Using a digital refractometer (Bellingham Stanley, DR103L, Tunbridge Wells, UK), the soluble solid content of the treated samples was determined and reported as ^o^Brix [35]. Using an Ultra-Turrax homogenizer (Daihan Scientific Co., HG-15 A, Wonju, Republic of Korea), 5 g of treated purslane samples were combined with 15 mL of distilled water. Following the addition of phenolphthalein, the homogenate was titrated with 0.1 M NaOH until the first persistent pale-pink color appeared. The titratable acidity was expressed as citric acid equivalents and calculated using Equation (3) [36].

acid% (wt/wt) = (Nx VxEq. wt./W × 1000) × 100(3)

N = Normality of titrant, NaOH (mEq/mL).
V = Volume of titrant (mL).
Eq. wt. = Equivalent weight of citric acid (mg/mEq).
W = mass of sample (g).
1000 = factor relating mg to grams (mg/g).

#### 2.11.3. Spectrophotometric Methods

- Extraction

5 g treated purslane samples were mixed with 25 mL of 95% ethanol solution using an Ultra-Turrax homogenizer (Daihan Scientific Co., HG-15 A, Wonju, Republic of Korea) at 6000 rpm for 2 min. The prepared mixtures were centrifuged (Sigma, 3–30 K) at 4000× *g* for 10 min. The supernatant was separated and stored at 4 °C until analysis.

- Total Flavonoid Content (TFC)

The procedure outlined by Viacava et al. (2018) was used to determine total flavonoid content (TFC) [37]. At room temperature, 1.28 mL of deionized water and 60 μL sodium nitrite solution (NaNO_2_, 5% *w*/*v*) were added to 0.2 mL of extracted samples. After 5 min, 60 μL of aluminum chloride (AlCl_3_, 10% *w*/*v*) solution was added to the samples. Samples were mixed with 400 μL sodium hydroxide (1 N NaOH) solution, after waiting another 6 min. Then, absorbance of samples was measured in a spectrophotometer (Thermo Scientific, Genesys 10S UVVIS, Madison, WI, USA) at 510 nm. Total flavonoid contents were expressed as mass of catechin equivalents per 100 g of fresh weight (mg CE/100 g FW) using a catechin calibration curve between 0.05 and 1 mg/mL.

- Total Phenolic Content (TPC)

The approach outlined by Viacava et al. (2018) was modified and used to measure the total phenolic content (TPC) of samples [37]. 200 μL of extracted samples were mixed with 1 mL of Folin–Ciocalteu reagent (1:10). After 3 min, 800 μL sodium carbonate (Na_2_CO_3_, 7.5% *w*/*v*) were added to obtained mixture and then mixing again. These steps occurred under room temperature. After 2 h of incubation, absorbance of samples was measured in a spectrophotometer at 765 nm. Total phenolic content was expressed as mass of gallic acid equivalents per 100 g of fresh weight (mg GAE/100 g FW) using gallic acid standard curve between 0.05 and 5 mg/mL.

- Chlorophyll content

2 g of treated purslane samples were crushed in a mortar by using 2 mL of 95% ethanol (*v*/*v*), periodically rinsed with 8 mL of 95% ethanol. After this process, samples were centrifuged for 10 min at 4000× *g* using a centrifuge (Sigma, 3–30 K, Osterode am Harz, Germany). The absorbances of the supernatants (A_663_ and A_645_) were measured spectrophotometrically at 663 and 645 nm. Chlorophyll a and chlorophyll b contents were calculated using Equations (4) and (5), respectively [38].

(4) $$Chlorophyll\;a,Ca\;(mg/g)=(12.72\times A_{663}-2.59\times A_{645})\times V/1000\;m$$

(5) $$Chlorophyll\;b,Cb\;(mg/g)=(22.88\times A_{645}-4.67\times A_{663})\times V/1000\;m\;\;\;\;\;$$

m = Purslane weight (g).
V = Volume of extracted sample (mL).

- Determination of antioxidant activity by using the DPPH assay

The method described by Ozdal et al. (2019) was used to determine the total antioxidant capacity (TAC) with a few modifications [39]. 0.1 mM DPPH (2,2-diphenyl-1-picrylhydrazyl) was prepared with 100 mL of ethanol. 2 mL of prepared DPPH solution was mixed with 100 μL of sample extract and shaken for 10 s. Then, the reaction mixtures were incubated in the dark. After 30 min., absorbance was measured using a spectrophotometer (Thermo Scientific, Genesys 10S UVVIS, Madison, WI, USA) at 517 nm. Total antioxidant capacity was expressed as mass of Trolox equivalents per 100 g of fresh weight (mg TE/100 g FW) using the Trolox standard curve between 0.05 and 0.5 mg/mL.

#### 2.11.4. Texture

The method was slightly modified from Irazoqui et al. (2019) [40]. Firmness (N) of samples was measured with a five-blade Kramer shear cell (HDP/KS5) fitted to a texture analyzer (Stable Micro Systems, TA.XT.plus, Godalming, UK). The pre-test, test, and post-test speeds were set to 2, 2, and 10 mm/s, respectively, while the other test parameters consisting of distance and target force were 30 mm, 5 kg respectively.

#### 2.11.5. Weight Loss

After washing treatments, all purslane samples were weighed as 33 g by using analytical balance (Ohaus, PA413C, Parsippany, NJ, USA), then stored at 4 °C along eight days. After each storage day (0th, 2nd, 3rd, 5th, 6th, 7th and 8th days), measurements were obtained from three independent biological replicates [41]. The weight loss (%) was calculated according to Formula (6).

(6) $$Weight\;loss\;\%=\frac{\left(Initial\;sample\;weight\;(0th\;day)-Sample\;weight\;at\;stored\;day\right)}{Initial\;sample\;weight\;(0th\;day)}\times 100$$

### 2.12. Statistical Analysis

All experiments were conducted using three independent biological replicates (*n* = 3) for each washing treatment. Each biological replicate consisted of a separately prepared purslane portion (100 g) independently subjected to the corresponding washing treatment. Analytical determinations were performed at least in duplicate as technical measurements (triplicate measurements for texture, weight loss, total phenolic content, and pesticide analysis; duplicate measurements for pH, °Brix, color, flavonoid, chlorophyll, DPPH, titratable acidity) within each biological replicate, and the technical replicates were averaged to obtain a single value for each independent experimental unit. Therefore, statistical analyses were based on three independent observations per treatment. Results are expressed as mean ± standard deviation. Prior to analysis of variance, normality of the residuals was assessed using the Shapiro–Wilk test and homogeneity of variances was assessed using Levene’s test. When these assumptions were satisfied, one-way analysis of variance (ANOVA) was performed, followed by Duncan’s multiple-range test was used to compare treatment means at *p* < 0.05. Statistical analyses were performed using IBM SPSS Statistics, Version 27.

## 3. Results and Discussion

### 3.1. Pesticide Residue Reduction in Purslane After Washing Treatments

**Figure 1:**
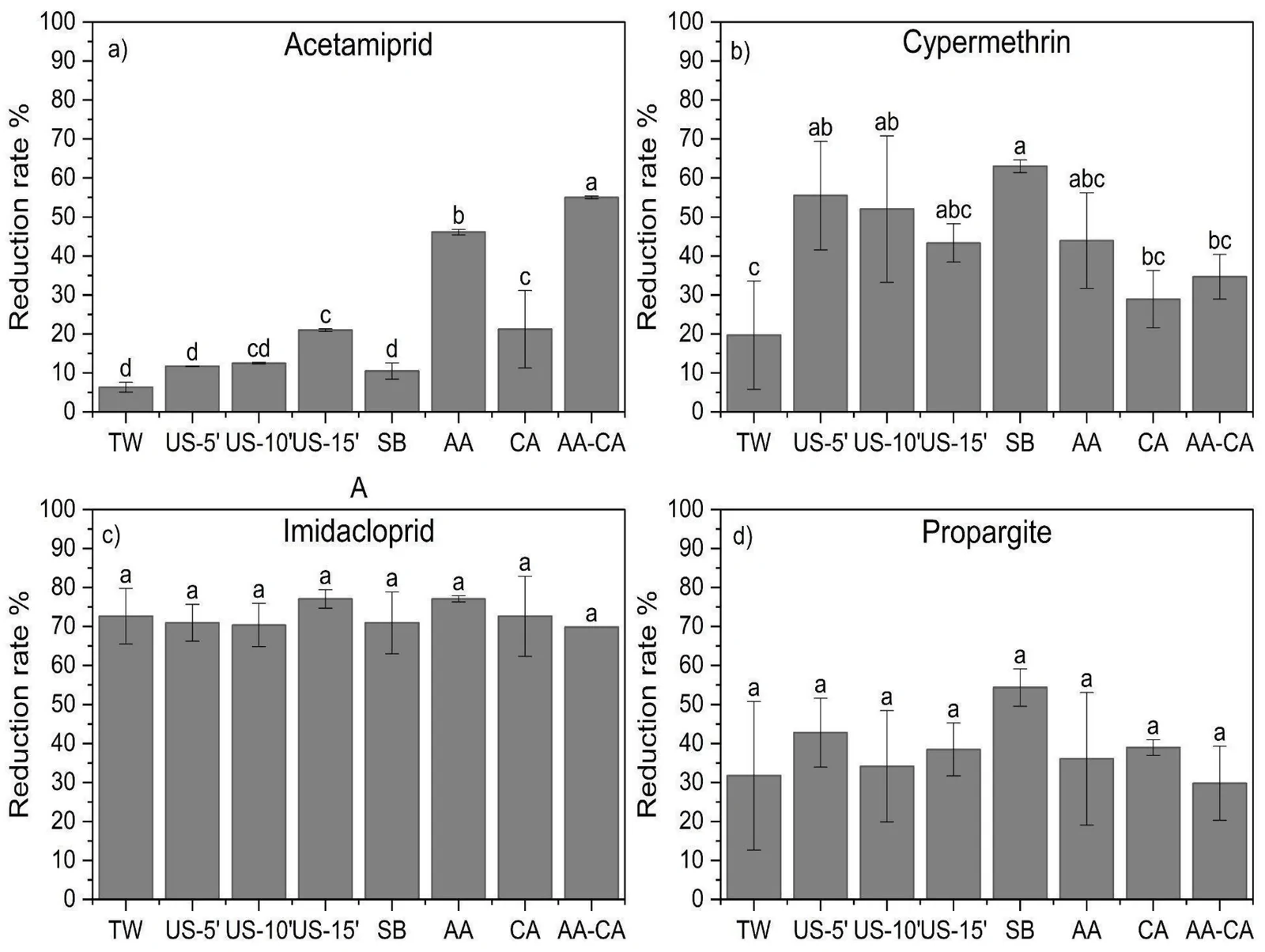
Effect of washing treatments on pesticide residue ((**a**) acetamiprid; (**b**) cypermethrin; (**c**) imidacloprid; (**d**) propargite) reduction in purslane (%).

Figure 1 shows the percentage reduction rates for different pesticide types in purslane samples after different washing treatments. According to the results, all washing treatments resulted in reductions of 70.00–72.22% and 27.00–52.00% for imidacloprid (Figure 1c) and propargite (Figure 1d), respectively. For imidacloprid, although its low log *P* and relatively high-water solubility would theoretically favor aqueous removal, no statistically significant differences among the washing treatments were detected in the present study (*p* > 0.05). Similarly, although propargite is highly lipophilic, statistically distinguishable differences among treatments were not observed (*p* > 0.05). These findings demonstrate that log *P* and water solubility alone cannot fully explain pesticide behavior during washing; pesticide formulation, surface deposition, cuticular interactions, systemic behavior, and treatment conditions may also contribute. These results do not demonstrate that all treatments were equivalent; rather, under the replication and variability of the present experiment, statistically distinguishable differences could not be established. The most effective washing solutions for reducing imidacloprid and acetamiprid in spinach were acetic acid, followed by citric acid and sodium carbonate [42]. Acetamiprid and imidacloprid are relatively hydrophilic pesticides, with log *P* values of approximately 0.8 and 0.57, respectively, and comparatively high-water solubilities. In contrast, cypermethrin and propargite are highly lipophilic compounds, with log *P* values of approximately 6.60 and 5.7, respectively, and very low water solubilities (Table A1). These differences may influence pesticide–cuticle interactions and the susceptibility of the residues to aqueous washing treatments. In the present study, the greatest reduction in acetamiprid was obtained with the acidic AA-CA treatment according to Figure 1a (*p* < 0.05). The relatively low log *P* and high-water solubility of acetamiprid may facilitate its transfer from the plant surface into an aqueous washing medium.

SB produced the highest numerical reduction in cypermethrin residues; however, its reduction did not differ significantly from those obtained with US-5′, US-10′, or US-15′ (Figure 1b). In addition, pesticide residue concentrations in untreated and washed samples are provided in Table A3. A combination of different concentrations of baking soda and ultrasound was reported to reduce fenpropathrin, cypermethrin, and deltamethrin without negative effect on the quality of the cabbage [43]. Applying efficient techniques that have less environmental impact and do not affect food quality is necessary for pesticide reduction [23]. In another study, the effects of different washing treatments on chlorpyrifos-methyl and lambda-cyhalothrin residues in Sultana grapes were investigated. It was determined that ultrasonic cleaning, tap water, acetic acid, citric acid treatments applied at different washing times and post-harvest intervals resulted in pesticide reductions of 13.9–62.8%, 19.5–66.4%, 28.0–66.7%, and 13.9–71.1%, respectively [44]. Physicochemical properties, processing methods, application time, and meteorological conditions during cultivation, and crop type influence the pesticide residue behavior [45].

The results confirmed that no single washing treatment was optimal for all four pesticides. Acidic solutions were more effective for acetamiprid, SB was more effective for cypermethrin, and statistically distinguishable treatment effects were not detected for imidacloprid and propargite. However, applying pesticides as acetonitrile-based standard solutions with only a 4 h holding time primarily reflects the reduction in freshly deposited surface residues. In addition, acetonitrile-based solutions may also alter the leaf wax layer, which can change how pesticides stick to or penetrate the leaves. In real fields, residues undergo weather conditions, plant uptake, and metabolism over longer periods. Because of this, pesticide residue reduction in commercial fields may be lower than the values observed in the experimental model used in this study.

### 3.2. Verification of Pesticide Extraction and Detection Methods

Method precision and recovery were evaluated using blank purslane samples spiked with acetamiprid, cypermethrin, imidacloprid, and propargite at a fortification level of 0.10 mg/kg in four replicates, with recovery and relative standard deviation values presented in Table 2. The obtained RSD and recovery values are presented in Table 2. Chromatographic performance assessment via LC-MS/MS and GC-MS/MS demonstrated method selectivity and linearity with correlation coefficients of 0.999 for all studied pesticides, while limits of detection and quantification are summarized in Table 2.

### 3.3. Effect of Washing Treatments on the Quality Characteristics of Fresh Purslane

One of the primary sensory qualities of fresh food is color, and consumers might not find it desirable if it changes from green to yellow to brown [38].

**Figure 2:**
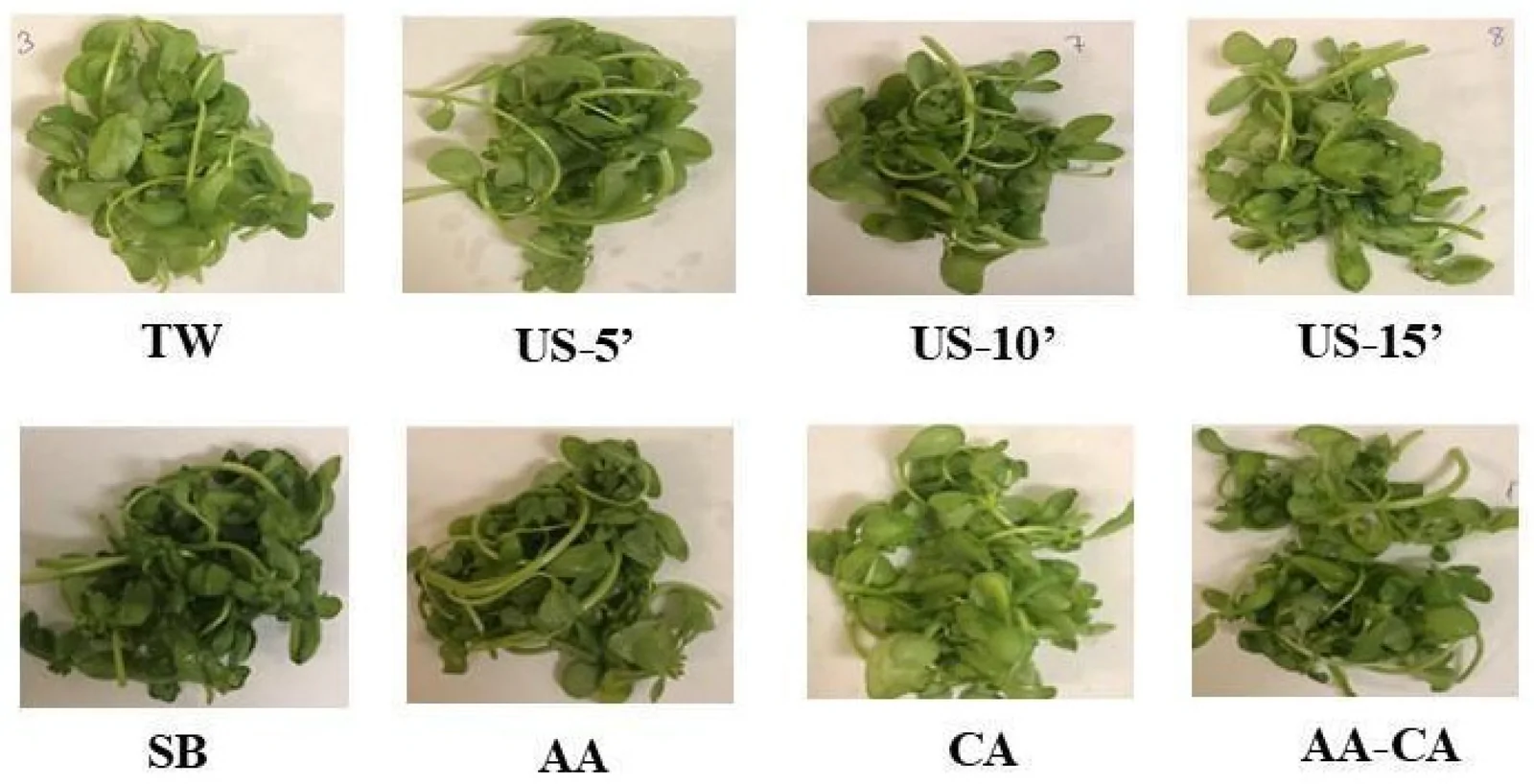
Appearance of purslane samples after washing treatments.

Table 3 presents the color parameters of purslane following the different washing treatments. The L* and b* values of the SB-treated samples were significantly lower than those of several other treatment groups (*p* < 0.05). No significant differences were observed among treatments for a* values (*p* > 0.05). Among the ultrasound-assisted washing treatments, b* values were 23.06 ± 2.76, 23.55 ± 2.21, and 21.60 ± 1.47 for US-5′, US-10′, and US-15′, respectively. Although US-15′ exhibited a numerically lower b* value, the differences among the three ultrasound treatment durations were not statistically significant (*p* > 0.05). Therefore, no consistent treatment-time-dependent trend in yellowness was evident within the investigated ultrasound treatment range. The change in color between the control and treated samples is represented by ΔE*, which may be further categorized as highly different (ΔE* > 3), distinct (1.5 < ΔE* < 3), and little difference (ΔE* < 1.5) [46]. The SB treatment exhibited a higher ΔE value (6.51 ± 0.91) than the other treatments. According to these parameters and Figure 2, these results indicate that 5% sodium bicarbonate solution caused the greatest change in the color and appearance of purslane. Although the AA treatment also exhibited a ΔE value greater than 3, no statistically significant difference was observed among the corresponding treatments (*p* > 0.05).

No significant differences were observed among US-5′, US-10′, and US-15′ for L*and ΔE*. Therefore, within the treatment range investigated, increasing ultrasound duration from 5 to 15 min did not produce a consistent or statistically significant treatment-time-dependent change in the measured color parameters.

According to the pH results (Table 4), SB exhibited the highest pH among the treatments. As expected, the acidic solutions (AA, CA, AA-CA) had the lowest pH values and highest TA% values. Although US-5′ resulted in a lower pH value, US-10′ and US-15′ resulted in higher pH values than TW (*p* < 0.05).

The SB sample had the lowest antioxidant activity, total phenolic content, and total flavonoid content values among all washing treatments (Table 4). The US-5′ and US-10′ washing treatments had the highest values (*p* < 0.05). The increase measured after short-duration ultrasound may have resulted from greater release or extractability of phenolic compounds following disruption of plant tissues. It should not be interpreted as evidence that ultrasound generated new phenolic compounds. Natural heterogeneity among purslane portions may also have contributed to the observed differences. Similarly, a previous study reported that *Vitis vinifera* leaves subjected to ultrasound for 5 and 10 min showed an increase in total phenolic content compared to the control sample [22]. In addition, it was determined that the combined AA-CA treatment significantly reduced flavonoid, total phenolic, and antioxidant activity compared to AA or CA applied individually (*p* < 0.05).

Consumers prefer firmness and crispness because they are associated with freshness and wholesomeness [47]. The firmness of fresh produce generally decreases during storage due to transpiration and respiration. Such changes can reduce product quality and accelerate deterioration [48]. Water loss negatively affects firmness and appearance and accelerates deterioration [49]. Among the washing treatments, US-10′ exhibited the greatest weight loss during storage and the lowest firmness. According to Table 4, there was no statistically significant difference in firmness values between the US-5′, AA-CA samples and the TW sample (*p* > 0.05). However, the remaining treatments showed significantly lower firmness than TW (*p* < 0.05).

Chlorophyll, which is chemically unstable, and sensitive to heat and light, can be used as an indicator of leafy-vegetable quality [28,50]. Weak acids, light, and oxygen promote chlorophyll degradation and the formation of degradation products [51]. The CA sample showed significantly lower chlorophyll B content compared to other samples (Table 4). In addition, The AA-CA sample has the lowest chlorophyll A value (*p* < 0.05).

**Figure 3:**
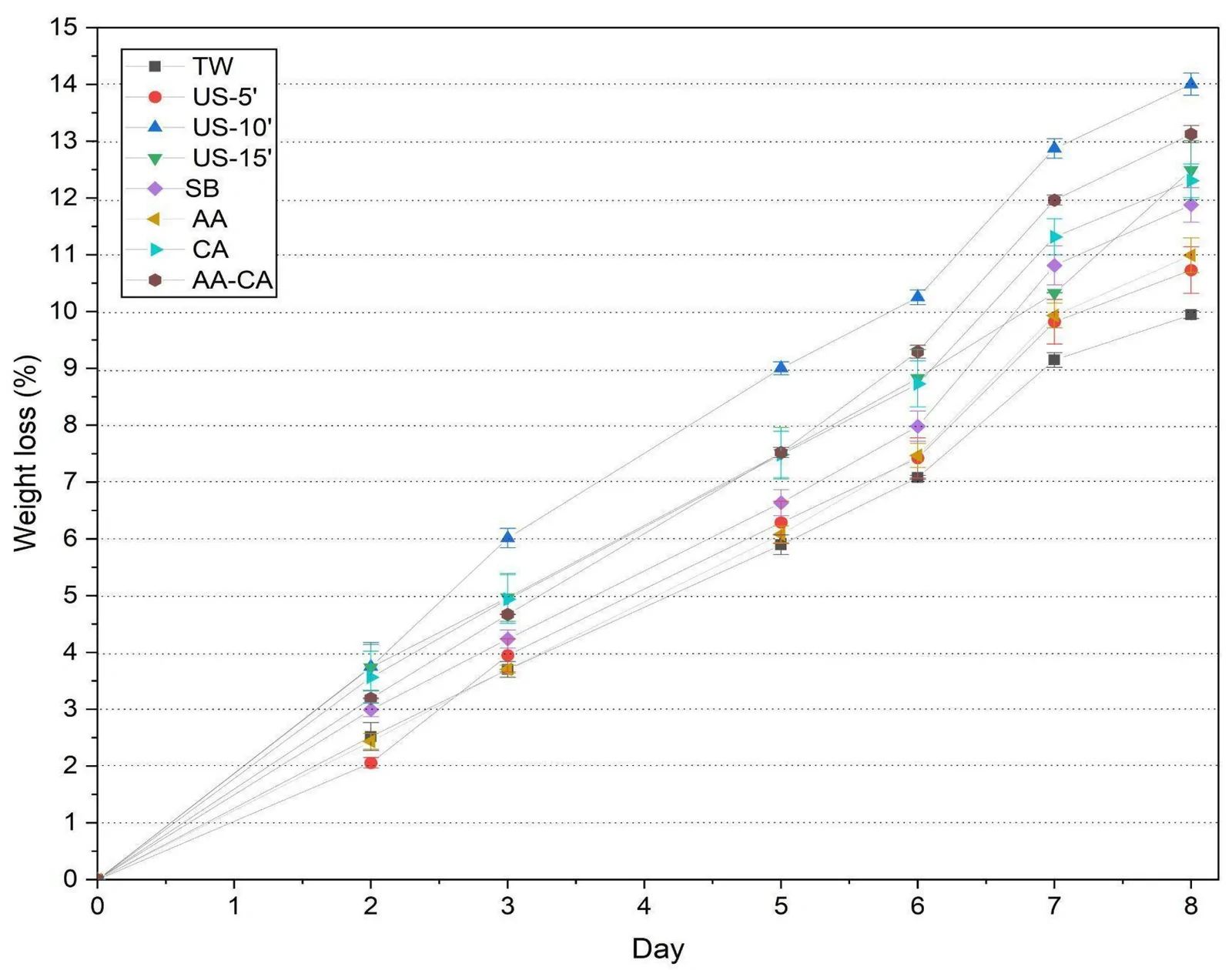
Weight loss (%) of purslane samples during storage.

Weight loss is an important indicator of freshness and postharvest quality in vegetables. In addition, it provides information on the product quality and shelf life [52]. According to Figure 3 and Table A4, in all washing applications, the weight loss % increased regularly as the storage time lengthened. From day 3 to the end of storage (including day 8), the highest weight loss values were statistically significantly measured in the US-10′ sample (*p* < 0.05). At day 8, TW exhibited the lowest weight loss (9.95 ± 0.07%), indicating the greatest retention of moisture content. In another study, different washing applications (5% sodium bicarbonate and 5% acetic acid) used for the reduction in pesticide residues in tomatoes resulted in a significantly greater weight loss compared to the control sample. At lower concentrations (1.5%), however, weight loss did not differ significantly from the control [53].

## 4. Conclusions

Monitoring pesticide residues in leafy vegetables is important for human health, food safety, and environmental protection. In this study, acetamiprid, cypermethrin, imidacloprid and propargite residues were analyzed in purslane samples after different washing treatments by LC-MS/MS and GC-MS/MS using the QuEChERS extraction method. Based on the pesticide-reduction results for imidacloprid and propargite, no statistically significant differences were detected between washing treatments. In addition, acidic treatments produced the greatest acetamiprid reduction. For cypermethrin, SB produced the highest reduction, although it did not differ significantly from the ultrasound-assisted washing treatments. However, SB and acidic treatments have negative effects on quality characteristics of samples. The antioxidant capacity and total phenolic content of SB samples were significantly lower than those of the other treatments. Color analysis showed significantly higher ΔE* values in SB samples compared to the control. The lowest chlorophyll B content was observed in the CA sample. In contrast, ultrasound-assisted washing, particularly the 5 min treatment, provided a favorable compromise between pesticide residue reduction and preservation of several quality attributes; however, the effects observed at longer ultrasound treatment times cannot be distinguished from those associated with prolonged immersion.

This study has several limitations. First, an artificial surface contamination model was utilized. Applying pesticides as acetonitrile-based standard solutions rather than commercial formulations may alter the leaf cuticular wax layer, affecting pesticide adhesion, penetration, and wash-off behavior. Furthermore, holding the spiked purslane for only 4 h simulates freshly deposited surface residues rather than field-aged contamination, where environmental weathering, translaminar uptake, and tissue binding occur. Consequently, these model-based reduction efficiencies cannot be directly extrapolated to commercial field conditions and may overestimate practical washing efficacy.

Second, the pesticides in the washing solutions and potential degradation products were not analyzed. Therefore, the observed decreases in residue concentration cannot be attributed specifically to physical transfer into the washing solution, adsorption to treatment vessels, chemical degradation, or redistribution within plant tissues. In addition, sensory acceptability, microbiological quality, and pilot-scale treatment performance were not investigated. Further studies involving naturally occurring residues or controlled pre-harvest applications, additional independent production batches, sensory evaluation, and pilot-scale ultrasound systems are needed to confirm the effectiveness under realistic agricultural conditions. In addition, future studies should evaluate ultrasound in combination with different washing solutions and monitor associated quality changes throughout storage.

## Figures and Tables

**Table 1:** Washing treatments of purslane samples.

Sample Code	Washing Treatments
TW	Tap water (5 min)
US-5′	Tap water (Ultrasound treatment, 5 min)
US-10′	Tap water (Ultrasound treatment, 10 min)
US-15′	Tap water (Ultrasound treatment, 15 min)
SB	5% sodium bicarbonate solution (5 min)
AA	5% acetic acid solution (5 min)
CA	5% citric acid solution (5 min)
AA-CA	2.5% citric acid + 2.5% acetic acid solution (5 min)

**Table 2:** Method validation data for studied pesticides.

Validation Parameters	Acetamiprid	Cypermethrin	Imidacloprid	Propargite
Linear range (mg/kg)	0.005–0.010–0.050–0.20	0.010–0.050–0.10–0.20	0.025–0.050–0.10–0.20	0.025–0.050–0.10–0.20
Calibration Equations	y = 383,089.322x + 612.942	y = 608,863x − 455	y = 56,190.626x + 410.283	y = 105,243x − 286
R^2^	0.999	0.999	0.999	0.999
LOD (mg/kg)	0.0014	0.0020	0.0079	0.0046
LOQ (mg/kg)	0.0041	0.0060	0.0239	0.014
Repeatability (RSD%)	1.39	2.46	1.47	2.74
Recovery %	101.40	97.94	102.16	102.54
Spike level (mg/kg)	0.10	0.10	0.10	0.10
Instrument	LC-MS/MS	GC-MS/MS	LC-MS/MS	GC-MS/MS

**Table 3:** Results of color parameters after washing applications for purslane samples.

Sample Code	L*	a*	b*	ΔE
TW	45.01 ± 1.66 ^a^	−7.43 ± 0.40 ^a^	22.44 ± 1.24 ^abc^	-
US-5′	45.08 ± 2.10 ^a^	−7.61 ± 0.57 ^a^	23.06 ± 2.76 ^abc^	2.41 ± 0.59 ^b^
US-10′	44.20 ± 1.87 ^a^	−7.55 ± 0.44 ^a^	23.55 ± 2.21 ^ab^	2.80 ± 0.78 ^b^
US-15′	44.87 ± 2.21 ^a^	−7.43 ± 0.80 ^a^	21.60 ± 1.47 ^bc^	2.47 ± 0.70 ^b^
SB	38.80 ± 0.98 ^b^	−6.96 ± 0.25 ^a^	20.63 ± 0.41 ^c^	6.51 ± 0.91 ^a^
AA	46.78 ± 1.14 ^a^	−7.58 ± 0.46 ^a^	24.94 ± 0.76 ^a^	3.26 ± 0.64 ^b^
CA	44.48 ± 1.50 ^a^	−7.53 ± 0.67 ^a^	23.48 ± 1.70 ^ab^	2.35 ± 0.20 ^b^
AA-CA	46.19 ± 1.95 ^a^	−7.28 ± 0.47 ^a^	23.14 ± 1.90 ^abc^	2.36 ± 1.66 ^b^

Different letters in the same column indicate a statistically significant difference between treatments (*p* < 0.05).

**Table 4:** Changes in quality characteristics of purslane samples after washing treatments.

Sample	TA (%)	pH	^o^Brix	Chlorophyll A (mg/g)	Chlorophyll B (mg/g)	Firmness (N)	Flavonoid (mg CE/100 g of Fresh Weight)	TPC (mg GAE/100 g of Fresh Weight)	DPPH (mg TE/100 g of Fresh Weight)
TW	0.38 ± 0.00 ^d^	5.17 ± 0.07 ^c^	1.80 ± 0.00 ^c^	90.69 ± 35.72 ^ab^	43.38 ± 6.77 ^ab^	13.19 ± 2.20 ^a^	210.68 ± 20.06 ^cd^	75.91 ± 8.56 ^ab^	74.63 ± 5.67 ^ab^
US-5′	0.35 ± 0.05 ^d^	5.05 ± 0.14 ^d^	1.68 ± 0.10 ^de^	84.93 ± 4.83 ^abc^	53.88 ± 26.10 ^ab^	11.48 ± 1.75 ^ab^	313.84 ± 7.11 ^a^	75.84 ± 7.53 ^ab^	82.18 ± 13.02 ^a^
US-10′	0.35 ± 0.05 ^d^	5.45 ± 0.10 ^b^	1.74 ± 0.13 ^cde^	93.67 ± 2.16 ^ab^	40.75 ± 5.95 ^ab^	8.54 ± 0.54 ^c^	252.66 ± 24.84 ^b^	78.21 ± 6.28 ^a^	74.08 ± 7.35 ^ab^
US-15′	0.39 ± 0.05 ^d^	5.52 ± 0.10 ^b^	1.85 ± 0.12 ^c^	108.44 ± 12.48 ^a^	43.52 ± 0.38 ^ab^	10.54 ± 0.77 ^bc^	227.77 ± 14.30 ^c^	76.08 ± 4.00 ^ab^	71.43 ± 13.08 ^ab^
SB	0.19 ± 0.06 ^d^	8.61 ± 0.06 ^a^	2.68 ± 0.08 ^a^	94.55 ± 47.20 ^ab^	59.72 ± 23.26 ^a^	9.41 ± 1.18 ^bc^	164.47 ± 20.82 ^e^	52.16 ± 13.76 ^d^	39.33 ± 4.86 ^c^
AA	2.10 ± 0.13 ^a^	4.02 ± 0.01 ^e^	1.78 ± 0.04 ^cd^	67.24 ± 4.92 ^abc^	41.57 ± 3.71 ^ab^	8.43 ± 0.92 ^c^	173.33 ± 16.75 ^e^	66.02 ± 7.67 ^bc^	80.57 ± 3.60 ^a^
CA	1.79 ± 0.19 ^b^	3.61 ± 0.01 ^f^	2.05 ± 0.05 ^b^	41.86 ± 12.56 ^bc^	22.08 ± 0.74 ^b^	9.68 ± 0.74 ^bc^	196.12 ± 8.06 ^d^	81.71 ± 3.91 ^a^	76.64 ± 7.99 ^ab^
AA-CA	1.13 ± 0.03 ^c^	4.12 ± 1.44 ^e^	1.63 ± 0.05 ^e^	35.09 ± 1.13 ^c^	35.31 ± 0.65 ^ab^	11.29 ± 2.02 ^ab^	123.65 ± 3.50 ^f^	57.04 ± 3.13 ^cd^	64.65 ± 14.88 ^b^

Different letters in the same column indicate a statistically significant difference between treatments (*p* < 0.05).

## Data Availability

The original contributions presented in this study are included in the article. Further inquiries can be directed to the corresponding author.

## Abbreviations

The following abbreviations are used in this manuscript:

AA	5% acetic acid (5 min)
AA-CA	2.5% citric acid + 2.5% acetic acid solution (5 min)
ANOVA	One-way analysis of variance
CA	5% citric acid solution (5 min)
CAS RN	Chemical Abstracts Service Registry Number
CE	Catechin equivalents
DPPH	2,2-diphenyl-1-picrylhydrazyl
ESI+/ESI-	Electrospray ionization source operating in positive and negative modes
FDA	Food and Drug Administration
FW	Fresh weight
GAE	Gallic acid equivalents
GRAS	Generally recognized as safe
IUPAC	International Union of Pure and Applied Chemistry
LOD	Limit of detection
log *P*	Logarithm of octanol-water partition coefficient
LOQ	Limit of quantification
MRLs	Maximum residue limits
MRM	Multiple Reaction Monitoring
p*K*_a_	Dissociation constant
RH	Relative humidity
RSD	Relative standard deviation
RTs	Retention times
SB	5% Sodium bicarbonate (5 min)
TA	Titratable acidity
TAC	Total antioxidant capacity
TE	Trolox equivalents
TFC	Total flavonoid content
TPC	Total phenolic content
TW	Tap water (5 min)
UAW	Ultrasound-assisted washing
US-5′	Tap water (Ultrasound treatment for 5 min)
US-10′	Tap water (Ultrasound treatment for 10 min)
US-15′	Tap water (Ultrasound treatment for 15 min)

## Appendix A

**Table A1:** Studied pesticides’ characteristics [54,55].

Name	IUPAC Name	CAS RN	Molar Mass (g/mol)	log *P* *	MRL for Purslane (mg/kg)	Solubility in Water	Structure
Acetamiprid	(E)-N1-[(6-chloro-3-pyridyl)methyl]-N2-cyano-N1-methylacetamidine	135410-20-7	222.67	0.80	0.6	High	C_10_H_11_ClN_4_, Insecticide Systemic
Propargite	2-(4-tert-butylphenoxy)cyclohexyl prop-2-ynyl sulphite	2312-35-8	350.7	5.70	0.01 *	Low	C_19_H_26_O_4_S, Acaricide, Insecticide Non-systemic
Cypermethrin	(RS)-α-cyano-3-phenoxybenzyl (1RS,3RS;1RS,3SR)-3-(2,2-dichlorovinyl)-2,2-dimethylcyclopropanecarboxy	52315-07-8	416.3	6.60	0.7	Low	C_22_H_19_Cl_2_NO_3_, Insecticide, Acaricide, Veterinary substance Non-systemic
Imidacloprid	(E)-1-(6-chloro-3-pyridylmethyl)-N-nitroimidazolidin-2-ylideneamine	138261-41-3	255.66	0.57	0.01 *	High	C_9_H_10_ClN_5_O_2_, Insecticide, Veterinary substance Systemic

* log *P* values were obtained from PubChem website [56].

**Table A2:** MRM transitions for selected pesticides.

Acetamiprid MRM transitions; ESI+		
Precursor *m*/*z*	Product *m*/*z*	CE
223	126.1	−20.0
223	56.2	−16.0
223	73.1	−55.0
Imidacloprid MRM transitions; ESI+		
Precursor *m*/*z*	Product *m*/*z*	CE
256.1	175.0	−20.0
256.1	209.0	−17.0
Cypermethrin MRM transitions; ESI−		
Precursor *m*/*z*	Product *m*/*z*	CE
163.0	127.0	5.0
163.0	91.0	10.0
181.1	152.1	22.0
181.1	127.1	22.0
Propargite MRM transitions; ESI+		
Precursor *m*/*z*	Product *m*/*z*	CE
368.2	231.1	−11.0
368.2	175.1	−17.0
368.2	57.1	−21.0

**Table A3:** Pesticide residues (mg/kg) for untreated and washing treatment applied samples.

Sample	Acetamiprid (mg/kg)	Cypermethrin (mg/kg)	Imidacloprid (mg/kg)	Propargite (mg/kg)
Inoculated (Untreated)	0.196 ± 0.003 ^a^	0.087 ± 0.001 ^a^	0.090 ± 0.001 ^a^	0.100 ± 0.006 ^a^
TW	0.183 ± 0.002 ^ab^	0.070 ± 0.012 ^ab^	0.025 ± 0.002 ^b^	0.071 ± 0.020 ^b^
US-5′	0.173 ± 0.000 ^b^	0.039 ± 0.012 ^de^	0.026 ± 0.004 ^b^	0.060 ± 0.009 ^b^
US-10′	0.171 ± 0.000 ^b^	0.042 ± 0.016 ^cde^	0.027 ± 0.002 ^b^	0.069 ± 0.015 ^b^
US-15′	0.155 ± 0.001 ^c^	0.049 ± 0.004 ^bcde^	0.026 ± 0.002 ^b^	0.064 ± 0.007 ^b^
SB	0.175 ± 0.004 ^b^	0.032 ± 0.001 ^e^	0.026 ± 0.003 ^b^	0.048 ± 0.005 ^b^
AA	0.106 ± 0.001 ^d^	0.049 ± 0.011 ^bcde^	0.026 ± 0.001 ^b^	0.067 ± 0.018 ^b^
CA	0.154 ± 0.019 ^c^	0.062 ± 0.006 ^bc^	0.025 ± 0.002 ^b^	0.064 ± 0.002 ^b^
AA-CA	0.088 ± 0.001 ^e^	0.057 ± 0.005 ^bcd^	0.027 ± 0.001 ^b^	0.073 ± 0.010 ^b^

Different letters in the same column indicate a statistically significant difference between treatments (*p* < 0.05). Initial acetamiprid concentration of purslane sample before inoculation process is determined as 0.095 mg/kg and initial concentration of other pesticides are not detected.

**Table A4:** Weight loss % results after washing treatments along storage days.

Sample	Day 2 (%)	Day 3 (%)	Day 5 (%)	Day 6 (%)	Day 7 (%)	Day 8 (%)
TW	2.52 ± 0.24 ^cde^	3.70 ± 0.14 ^d^	5.89 ± 0.17 ^d^	7.08 ± 0.04 ^d^	9.15 ± 0.13 ^f^	9.95 ± 0.07 ^e^
US-5’	2.06 ± 0.09 ^e^	3.95 ± 0.29 ^d^	6.29 ± 0.37 ^cd^	7.42 ± 0.35 ^cd^	9.83 ± 0.39 ^e^	10.73 ± 0.41 ^d^
US-10’	3.73 ± 0.42 ^a^	5.99 ± 0.17 ^a^	8.98 ± 0.11 ^a^	10.23 ± 0.13 ^a^	12.85 ± 0.17 ^a^	13.98 ± 0.20 ^a^
US-15’	3.73 ± 0.40 ^a^	4.96 ± 0.42 ^b^	7.50 ± 0.45 ^b^	8.82 ± 0.50 ^b^	10.32 ± 0.01 ^de^	12.49 ± 0.52 ^bc^
SB	3.00 ± 0.12 ^bcd^	4.24 ± 0.16 ^cd^	6.64 ± 0.23 ^c^	7.99 ± 0.27 ^c^	10.82 ± 0.34 ^cd^	11.89 ± 0.30 ^c^
AA	2.43 ± 0.14 ^de^	3.70 ± 0.07 ^d^	6.08 ± 0.15 ^cd^	7.47 ± 0.21 ^cd^	9.93 ± 0.22 ^e^	10.99 ± 0.31 ^d^
CA	3.57 ± 0.46 ^ab^	4.94 ± 0.43 ^b^	7.49 ± 0.41 ^b^	8.73 ± 0.40 ^b^	11.32 ± 0.32 ^c^	12.31 ± 0.30 ^c^
AA-CA	3.19 ± 0.01 ^abc^	4.67 ± 0.01 ^bc^	7.52 ± 0.09 ^b^	9.29 ± 0.12 ^b^	11.96 ± 0.09 ^b^	13.13 ± 0.15 ^b^

Different letters in the same column indicate a statistically significant difference between treatments (*p* < 0.05).
